# Effects of myo-inositol plus alpha-lactalbumin in myo-inositol-resistant PCOS women

**DOI:** 10.1186/s13048-018-0411-2

**Published:** 2018-05-10

**Authors:** Mario Montanino Oliva, Giovanna Buonomo, Marco Calcagno, Vittorio Unfer

**Affiliations:** 1 Department of Woman Health and Reproductive Medicine, Santo Spirito Hospital, 00193 Rome, Italy; 2 Faculty of Medicine and Psychology, Department of Developmental and Social Psychology, Sapienza University, 00185 Rome, Italy

**Keywords:** Myo-inositol, Alpha-lactalbumin, Polycystic ovary syndrome, Anovulation, Infertility

## Abstract

**Background:**

Myo-inositol (MI), successfully used in polycystic ovary syndrome (PCOS), was administered with α-LA to exploit its action of favouring the passage of other molecules through biological barriers, and also considering its anti-inflammatory effect.

**Methods:**

PCOS patients, according to the Rotterdam ESHRE–ASRM criteria, with anovulation and infertility > 1 year, were included in this open and prospective study. The preliminary phase was aimed at determining a set of MI-resistant PCOS patients. This treatment involved 2 g MI, taken twice per day by oral route, for three months. The Homeostasis Model Assessment (HOMA) index and MI plasma levels were measured. In the main phase, previously selected MI-resistant patients received the same daily amount of MI plus 50 mg α-LA twice a day, for a further three months. Ovulation was assessed using ultrasound examination on days 12, 14 and 20 of the cycle. The HOMA index, lipid, hormone and MI plasma levels were detected at baseline and at the end of this phase.

**Results:**

Thirty-seven anovulatory PCOS subjects were included in the study. Following MI treatment, 23 of the 37 women (62%) ovulated, while 14 (38%) were resistant and did not ovulate. In the latter group, MI plasma levels did not increase. These MI-resistant patients underwent treatment in the main phase of the study, receiving MI and α-LA. After this combined treatment, 12 (86%) of them ovulated. Their MI plasma levels were found to be significantly higher than at baseline; also, a hormone and lipid profile improvement was recorded.

**Conclusion:**

The combination of MI with α-LA allowed us to obtain significant progress in the treatment of PCOS MI-resistant patients. Therefore, this new formulation was able to re-establish ovulation, greatly increasing the chances of desired pregnancy.

**Trial Registration:**

Clinical trial registration number: NCT03422289 (ClinicalTrials.gov registry).

## Background

The diagnostic traits of PCOS, according to the 2003 Rotterdam criteria, require at least two of the following clinical and endocrine features: chronic ovulatory disorder, clinical and/or biochemical hyperandrogenism and polycystic ovaries [1]. Among these criteria, insulin resistance (IR) is not included; however, deregulation of insulin sensitivity, and/or abnormalities in glucose metabolism, may be found in many PCOS patients, linked to obesity (or not) [2]. It is estimated that up to 80% of PCOS women with upper-body obesity show IR, whereas 30–40% of PCOS lean women are affected by hyperinsulinemia [3]. Currently, IR and/or compensatory hyperinsulinemia account for important disorders implicated in PCOS [4]. Changes in diet and lifestyle can improve ovary function and prevent later PCOS-related risks in some patients; however, in most cases differentiated therapies with drugs and/or nutritional supplements are mandatory [5]. In the last few years, two stereoisomers of inositol, myo-inositol (MI) and D-chiro-inositol (DCI) have attracted scientific interest and related research on them has gained momentum. MI and DCI are cyclic polyols (C_6_H_12_O_6_) with MW 180.16, present in all living forms; they are involved in a wide array of metabolic pathways with significant therapeutic applications in pathologies (e.g., PCOS, GDM, MetSyn) where insulin sensitizing agents play a key role [4, 6, 7]. Obviously, they are even more useful as they do not involve significantly adverse events [8]. Among their manifold physiological activities, we highlight that in the ovary MI regulates glucose uptake and FSH signalling, while DCI modulates insulin-induced androgen synthesis [9]. Three different human studies carried out in patients undergoing IVF showed that MI plays a very beneficial role on oocytes, whereas an increase of DCI is detrimental [10–12]. It was demonstrated that effects of MI are pivotal in counteracting important endocrine and metabolic anomalies related to PCOS. MI was seen to be effective in restoring spontaneous ovarian activity and, consequently, promoting fertility in most patients with PCOS. For instance, oral treatment with 2 g MI twice a day was clinically investigated in several trials and is now considered one of the standard treatments for PCOS [13]. Despite this, as occurs with pharmacological treatments (e.g. metformin, clomiphene citrate, etc.), MI is not always completely effective in treating PCOS or associated conditions such as anovulation, fertility and subfertility in all patients. This could be related to varying individual response to the same therapy. Indeed, Kamenov et al. [14] reported that while treating 50 anovulatory PCOS women with 4 g/day MI still resulted in 38.3% of MI-resistant patients (i.e. in women who did not ovulate even after the therapy). Thus, while the important role of MI in human reproduction is known and consequently MI supplementation has been proposed as a reliable treatment for women affected by PCOS, there is still a significant room for intervention to improve the treatment of infertility or subfertility, in particular relating to PCOS or anovulation, in the MI-resistant group. A reason for this MI-resistance may be due to insufficient MI absorption in this group of patients.

A molecule such as α-lactalbumin (α-LA) may offer the solution to overcome this problem. α-LA is a milk protein found in all mammals. and is the second most abundant protein (from 20 to 25%) in whey [15], with low immunogenicity in comparison to other allergens in cow’s milk [16]. It shows a wide array of therapeutic effects, and plays numerous roles in addition to its nutritional function; we call attention to the increase in the passage of steroids or metals through biological membranes (i.e. vitamin D, iron) [17, 18], the stimulation of growth and the modulation of the immune system [19].

The aim of this study was to test a new formulation made up of MI and α-LA to successfully treat MI-resistant PCOS women.

## Methods

### Patients

In this open and prospective study, patients were assessed for eligibility from November 2016 to March 2017 at the Department of Women’s Health and Reproductive Medicine (Santo Spirito Hospital, Rome, Italy).

Inclusion criteria were: age 20–35 years; PCOS according to the Rotterdam ESHRE–ASRM consensus workshop group [1]; anovulation and infertility > 1 year. Further, according to the Rotterdam criteria, PCOS was diagnosed if 2 out of the 3 following conditions were met: a) oligo- or anovulation, b) clinical and/or biochemical signs of hyperandrogenism, and c) polycystic ovaries.

Exclusion criteria were: the presence of other pathologic conditions causing ovulatory dysfunction (such as hyperprolactinemia or hypothyroidism), or androgen excess (such as adrenal hyperplasia or Cushing’s syndrome); the intake of other drugs that could potentially influence ovulation. Also, obese women were excluded, as well as women with partners with sperm abnormalities. In this way, we ruled out the concomitant factors that interfere with the possibility of becoming pregnant as potentially confounding variables.

Clinical trial registration number: NCT03422289 (ClinicalTrials.gov registry).

This trial has a preliminary and a main phase:

1. the preliminary phase was aimed at establishing a set of MI-resistant PCOS patients;
2. the main phase focused on verifying whether the addition of α-LA to MI can allow successful treatment in MI-resistant women

The primary outcome was restoring ovulation in MI-resistant PCOS patients; a secondary outcome was evaluating the increase of MI plasma levels from baseline to end of the new therapy in MI-resistant patients.

### Treatments and patient evaluation

In the preliminary phase, all the patients were given 2 g MI in powder form, twice a day by oral route before meals, for three months, in order to induce ovulation. The treatment of infertility or subfertility, particularly infertility or subfertility caused by PCOS or anovulation, as well as related to the treatment of PCOS or anovulation itself, can be achieved in the majority of patients with MI alone. In the main phase of treatment, MI-resistant subjects (selected in the preliminary phase of therapy) received 2 g MI plus 50 mg α-LA in powder form, twice a day by oral route before meals for three months.

Ovulation was assessed using ultrasound examination on days 12, 14 and 20 of the cycle.

The Homeostasis Model Assessment (HOMA) Index and MI plasma levels were evaluated in all the patients in the first and second phase of therapy.

A panel of lipids and hormones, as well as MI, were measured in plasma of all the patients enrolled in the second cycle of therapy. These assays were made at baseline and at end of the treatment. Blood samples were centrifuged when obtained, and immediately stored at 20 °C until assayed.

### Assays in plasma

All the analyses were performed using commercial kits, except for MI. Insulin levels were assessed using a DPC Immulite 2000 analyser (Euro/DPC, Llanberis, UK). The presence of IR was established by means of the HOMA index. Cholesterol was measured with an enzymatic cholesterol oxidase/peroxidase method (Beckman Coulter Diagnostics, Brea, CA, USA), and triglycerides were measured with an enzymatic assay (Beckman Coulter Diagnostics, Brea, CA, USA); HDL-cholesterol test and LDL-cholesterol test were performed (Beckman Coulter Diagnostics, Brea, CA, USA); progesterone, testosterone and SHBG with an ECLIA (Electrochemiluminescence immunoassay) kit (Roche Diagnostics, Mannheim, Germany); 17β-estradiol with a competitive immunoassay (Access Immunoassay System, Estradiol, Beckman Coulter, Brea, CA, USA); DHEAS with an enzymatic assay (Roche Diagnostics, Mannheim, Germany). Free testosterone and androstenedione were measured with RIA kit (Beckman Coulter Diagnostics, Brea, CA, USA).

MI levels were assayed in accordance with the following procedure. Firstly, extraction with organic solvents and derivatization were performed, then samples were analysed by gas chromatography-mass spectrometry with Agilent 6890 (Agilent, 5301 Stevens Creek Blvd, Santa Clara, CA 95051, USA). A volume of 1.0 μl was injected in a split-less mode at 270 °C, using a capillary column Agilent 122–5532 dB-5 ms (0.25 mm × 30 m × 0.25 μm). The total run-time lasted 15 min: an oven at 70 °C from 0 to 1 min; 20 °C/min to 150 °C; 10 °C/min to 240 °C; 4 min at 320 °C post-run. Flow rate:1.2 ml/min. The results were analysed by a MS 5973 Network Series detector in sim mode. MI levels were expressed in μmol/l.

### Statistical analysis

The results are displayed as means ± standard deviation (SD). Lipid and hormone parameters were evaluated by comparing the changes from baseline to the end of the trial in the treated group with the corresponding changes in controls. The changes were compared using Student’s two-tailed unpaired t-test.

## Results

Thirty-seven anovulatory PCOS patients were included in the study. Their features at baseline are shown in Table 1.

**Table 1:** Baseline characteristics of enrolled patients (no. = 37)

Age (years)	27.1 ± 2.4
BMI (kg/m^2^)	25.9 ± 1.2
HOMA Index	5.51 ± 1.9

Data presented as mean ± SD

Following MI treatment for three months, 23 of the 37 women (62%) ovulated, while 14 (38%) did not ovulate. They failed to respond to MI treatment and for this reason were classified as MI-resistant. BMI did not change in responder and non-responder patients (the new means ± SD were 25.6 ± 1.7 and 25.8 ± 1.9, respectively, vs 25.9 ± 1.2 at baseline), whereas the mean ± SD of HOMA Index decreased to 3.3 ± 1.1 in responders and was fundamentally unchanged in MI-resistant subjects (5.44 ± 2.0), compared to 5.51 ± 1.9 at baseline.

These 14 MI-resistant patients were included in the second phase of the study, where they were administered orally with 2 g MI plus 50 mg α-LA, twice a day, for the following three months. Among these 14 subjects, 12 (86%) ovulated. Their MI plasma levels at the end of the treatment significantly improved compared to the baseline (35.0 ± 3.8 μmol/l versus 17.0 ± 3.5 μmol/l, the latter having been measured at the beginning of the second phase of the study) and were similar to the patients who responded positively to the treatment with MI alone (38 ± 2.9 μmol/l) (Table 2). Therefore, an increase in MI absorption was related to the restoration of ovulation.

**Table 2:** Ovulation results and MI plasma concentration in patients undergoing the first and the second phase of the study

	MI plasma levels		Ovulation
	T0 μmol/l	T3 μmol/l	T3 (n)
First phase: treatment with MI alone for 3 months in all patients (no. = 37)	–	38 ± 2.9	23/37
Second phase: treatment with MI + α-LA for 3 (further) months in MI-resistant women (no. = 14)	17 ± 3.5^a^	35 ± 3.8^b^	12/14

Values are expressed as mean ± SD. *T0* baseline; *T3* end of treatment (three months)

^a^this value was found in MI-resistant subjects in the first phase after MI treatment (T3) and represents the baseline value of the second phase

^b^MI plasma levels in MI-resistant women at T3 were found to be significantly (*p* <  0.001) higher than at T0

Furthermore, responders to the therapy showed an improvement in the following parameters, consistent with restored ovulation: total cholesterol, triglycerides, testosterone, free testosterone, androstenedione, dehydroepiandrosterone sulfate, sex hormone-binding globulin (SHBG). The improvement was significant for all the parameters cited above, except for androstenedione (Table 3).

**Table 3:** Lipid and hormone plasma levels in MI-resistant patients at baseline and after MI plus α-LA treatment

Plasma levels of:	Baseline	End of treatment	*p*
Total cholesterol (mg/dl)	211 ± 22	190 ± 26	≤ 0.05
HDL (mg/dl)	35 ± 8	37 ± 6	n.s.
LDL (mg/dl)	128 ± 21	127 ± 20	n.s.
Triglycerides (mg/dl)	186 ± 51	119 ± 38	≤ 0.05
Progesterone (ng/ml)	0.7 ± 0.3	0.6 ± 0.1	n.s.
Testosterone (ng/dl)	88 ± 38	57 ± 31	≤ 0.05
Free testosterone (ng/dl)	1.3 ± 0.5	0.6 ± 0.3	< 0.001
Androstenedione (ng/dl)	198 ± 59	165 ± 47	n.s.
17β-estradiol (ng/dl)	9.1 ± 3.7	9.2 ± 3.9	n.s.
Dehydroepiandrosterone sulfate (μg/dl)	530 ± 231	279 ± 87	< 0.01
SHBG (μg/dl)	2.6 ± 0.9	5.0 ± 2.1	< 0.001

Values are expressed as mean ± SD. *p*: value at end of treatment vs baseline

Instead, no significant changes were detected in the two MI-resistant women (data not shown)

## Discussion

As the above results clearly indicate, the combination of MI with α-LA allows a significant improvement in PCOS treatment in MI-resistant patients. This combination was able to re-establish ovulation, together with a consistent variation in metabolic and hormone parameters, thus greatly increasing the chances of a desired pregnancy in women who would otherwise have remained infertile. These effects may be related to an increase in MI daily plasma levels in the patients who then ovulated. We have to stress that in a study by Raffone et al. [20] the period of treatment with myo-inositol lasted six months; however, it did not reduce the number of MI-resistant patients, obtaining a value (35%) that was even slightly lower than that found by our team after three months.

Furthermore, we should take in account an effect directly exerted by α-LA on PCOS (we will discuss this point later). Our results strongly increase the possibility of treating this subset of patients who so far have been MI-resistant. Furthermore, this combination could open up new prospects for people affected by other disorders (i.e. MetSyn and GDM) where MI was administered with very promising results in many patients but not all [7]. Our data demonstrate that this improvement is due to the presence of α-LA. In this study, patients were administered twice a day for three months with 2 g MI and 50 mg α-LA. The substantial length of this treatment allows us to speculate on some different pathways through which α-LA may achieve its effects.

We have to remember that some mechanisms are believed to maintain MI concentrations in tissues and cells and work at different levels: intestinal uptake and kidney excretion, transport from plasma into cells, endogenous synthesis and catabolism. Active processes against the concentration gradient are involved in the transporting of MI into numerous cell types, taking advantage of two types of carrier (sodium-coupled and proton-coupled) [21–23]. It allows higher intracellular concentrations to be reached compared to outside the cells, i.e. plasma or culture medium. With specific reference to the current study, the low Km (50–300 μM) of both Na+/linked myo-inositol transporters and H+/myo-inositol transporter [21–23] suggest the involvement of other mechanisms. In fact, MI can achieve quite high concentrations in the gut, which are compatible with the administered oral dose [18]. A personal communication by the authors (G. Monastra, Y. Sambuy; S. Ferruzza; D. Ferrari, G. Ranaldi) of a recent still unpublished in vivo and in vitro study informed us that the simultaneous administration of MI and α-LA leads to a significantly higher bioavailability of MI than observed when MI is given by itself. This new formulation was found to be very promising and may have several therapeutic applications which is why there is a patent pending aiming at covering this. The authors have seen that MI bioavailability improved when this molecule was administered along with α-LA to healthy volunteers. In these subjects, a single oral dose of MI and α-LA was able to raise MI plasma concentrations in a significant way (32.4%), when compared to the administration of MI alone. Similarly, a three-fold increase in MI transport was seen in Caco-2 cell cultures when MI was added to the biopeptides coming from in vitro α-LA digestion that mirrors what occurs in vivo to α-LA when it transits through the stomach and upper small intestine. The cause of this higher passage was attributed to a physiological increase in the permeability of tight junctions. However, this mechanism cannot exclude other complementary possibilities of improving MI transport, when treatment lasts for weeks. In this case, another hypothesis might involve the promotion of glucagon-like peptide-2 (GLP-2) secretion from gut endocrine cells by α-LA hydrolysate which was observed in rats [24]. The increase in GLP-2 levels was proven to enhance small intestine absorption in normal rats in vivo after a 14-day administration of this peptide by means of jugular venous catheters. GLP-2 acts as a growth factor to expand the surface area of the mucosal epithelium and increases molecule transport in enterocytes. It was found that this peptide significantly stimulates galactose and glycine absorption [25]. The same effect might occur with MI. Furthermore, α-LA, in specific conditions, can work as a suitable carrier to increase the passage of molecules across the intestinal barrier. In an in vitro study [17], binding properties and conformational change of bovine apo α-LA upon interaction with vitamin D3 were investigated by calorimetry, spectroscopy and by molecular docking. The authors stated that vitamin D3 takes advantage of having a binding site on α-LA to enhance bioavailability. It is worthwhile noting that although the particle size of the complex formed between α-LA and vitamin D3 is much larger than the native protein, this steric increase does not hamper vitamin D3 transfer [17]. Another study carried out on healthy-term, six-month-old children showed that α-LA does not exert any effect on iron absorption from infant formula [26]; however, an in vitro study with Caco-2 cells demonstrated that α-LA hydrolysate-Fe complex might improve iron absorption compared with the mixture of α-LA hydrolysate and iron [18]. Therefore, α-LA would appear to be a carrier that is equipped with specificity towards selected molecules and in specific conditions. Even though the data available nowadays concern a secosterol and iron, it may be possible that also in our study α-LA was able to bind MI, thus facilitating its passage through the intestinal barrier. In the last decade, new findings have shown evidence of other factors playing a crucial role in the onset and maintenance of PCOS, giving a wider insight on this very complex and multifaceted pathology. Also, these new aspects might be related to α-LA activity found in our study. Two well-established hypotheses have emerged on the aetiopathogenesis of this syndrome, which refer to the alteration of the intestinal microbiota [27, 28] as well as the presence of a chronic inflammatory state [29, 30]. It has been shown that the microbiota of PCOS patients is altered in terms of species and the related number of strains, compared to that of healthy subjects. This dysbiosis of gut microbiota is believed to play a key role in triggering important processes underlying PCOS [27, 28]. An imbalance among the respective percentages of bacterial strains appears to generate several alterations in organism physiology, not only with reference to the absorption of some nutrients [31] but also, and above all, in determining and maintaining a chronic inflammatory process [28, 31]. On the other hand, inflammation has been recently defined as an important cause of PCOS [29, 30]. A systemic chronic inflammatory state, found in numerous patients with PCOS, appears to be the main cause of the traits related to this pathology, such as hyperandrogenism and IR Inflammation in women with PCOS (which appears to be established by multiple factors among which, as reported before, intestinal dysbiosis) [28]. Furthermore, a chronic inflammatory state in PCOS may have, in some cases, a genetic origin. In fact, in subjects with this syndrome, there are some functional alterations in the genes involved in the synthesis of interleukin-6 (IL-6) and tumor necrosis factor-α (TNF-α) which induce a greater release of these cytokines [30]. Initially, it was believed that hyperandrogenism was caused only by hyperinsulinemia due to the compensatory effect caused by IR. However, it has been recently put forward that hyperandrogenism, as well as being induced by insulin, may be directly induced by inflammation, regardless of the presence of IR [29, 32, 33]. The presence of an altered gut microbiota and of a chronic inflammation may explain other possible effects exerted by α-LA. As mentioned earlier, we know that an imbalanced microbiota can hamper the absorption of a wide array of substances. In such contexts, it is highly plausible that the beneficial changes in the composition of gut microbiota due to α-LA therapeutic activity after its prolonged administration can improve MI bioavailability. This rebalancing of gut flora may well promote a better uptake of a substance such as MI. To our knowledge, very few studies on the possible effects of α-LA in this field have been carried out, on experimental animals or humans (only new-borns, not adults). Among them, there is a patent on α-LA as a prebiotic agent referencing data found with rats [34]. We can also quote a study on a milk formula for infants, enriched in α-LA and administered for months, which can improve the growth of specific bacterial groups in healthy-term infants in the same way breast milk does [35]. In addition, α-LA is an anti-inflammatory molecule, endowed with the capacity to inhibit type 2 cyclooxygenase (COX 2) and to decrease the inflammatory cytokine IL-6 [36]. In addition, α-LA was able to decrease blood glucose levels after glucose loading in a rat model of type 2 diabetes [37]. Therefore, α-LA per se may strongly reduce the chronic inflammation connected to PCOS, also with reference to IR, thus cooperating with MI’s healing activity.

An important connotation of our study is also the prospect of adopting the treatment with MI plus α-LA in IVF, a procedure where time-saving is crucial. In fact, our data agree with those produced by Kamenov and demonstrate that there is a set of patients that are MI-resistant and therefore they do not ovulate after MI administration for three months. Clearly, the small number of patients included represents a limitation of this study, however the new treatment containing MI plus α-LA would allow us to save three months of therapy and, based on our evidence, it could be the first line therapy for all PCOS women undergoing IVF.

## Conclusion

The results obtained demonstrate that the administration of MI in combination with α-LA represents significant therapeutic progress in the field of PCOS. Obviously, these results need to be confirmed with a wider sample size, by means of randomized controlled trials. However, they already provide compelling evidence that supports this new treatment for MI-resistant patients.

## Abbreviations

α-LA: α-lactalbumin
BMI: Body mass index
COX 2: Type 2 cyclooxygenase
DCI: D-chiro-inositol
FSH: Follicle-stimulating hormone
GDM: Gestational diabetes mellitus
GLP-2: Glucagon-like peptide-2
HOMA: Homeostasis Model Assessment
IL-6: Interleukin-6
IR: Insulin resistance
IVF: In vitro fertilization
MetSyn: Metabolic syndrome
MI: Myo-inositol
MII: Metaphase II stage
PCOS: Polycystic ovary syndrome
rFSH: recombinant Follicle-stimulating hormone
SHBG: Sex hormone-binding globulin
TNF-α: Tumor necrosis factor-α.
